# Current strategies to treat tuberculosis

**DOI:** 10.12688/f1000research.7403.1

**Published:** 2016-10-26

**Authors:** Anthony T. Podany, Susan Swindells

**Affiliations:** 1 College of Pharmacy, University of Nebraska Medical Center, Omaha, Nebraska, USA; 2 Department of Internal Medicine, University of Nebraska Medical Center, Omaha, Nebraska, USA

**Keywords:** Tuberculosis, Rifamycin, Fluoroquinolones, Clofazamine, Bedaquiline, Nitroimidazoles, Pretonamid, Delamanid, Oxazolidinones

## Abstract

Tuberculosis (TB) has been a leading cause of death for more than a century. While effective therapies exist, treatment is long and cumbersome. TB control is complicated by the overlapping problems created by global inadequacy of public health infrastructures, the interaction of the TB and human immunodeficiency virus (HIV) epidemics, and the emergence of drug-resistant TB. After a long period of neglect, there is now significant progress in the development of novel treatment regimens for TB. Focusing on treatment for active disease, we review pathways to TB regimen development and the new and repurposed anti-TB agents in clinical development.

## Introduction

In 2014, the World Health Organization (WHO) reported 9.6 million incident cases of tuberculosis (TB), 1.1 million deaths from TB among human immunodeficiency virus (HIV)-negative people, and an additional 0.4 million deaths from HIV-associated TB ^1^. TB is the fifth leading infectious killer of adults worldwide ^2^, the third largest killer of women in their reproductive years ^3^, and a leading infectious cause of death among people with HIV disease ^2– 5^. In recent years, the world has seen a rapidly emerging epidemic of drug-resistant TB, multi-drug-resistant TB, and extensively drug-resistant TB, which is lethal and extremely expensive and complicated to treat ^6^.

**Figure 1.**
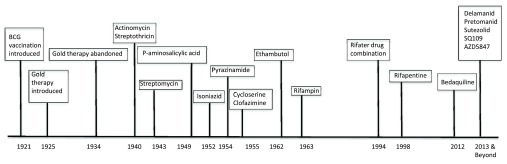
Timeline for tuberculosis (TB) drug development. Major milestones in TB drug development over the last century are outlined by the date of approval of the drug or intervention. Approval dates for drugs in development are estimated. Abbreviations: BCG, Bacillus Calmette-Guérin.

The current first-line four-drug regimen for drug-susceptible TB is nearly 50 years old, takes six to nine months to complete, and has significant side effects. Treatment for drug-resistant TB may take up to 30 months ^7^. Even though more than 250,000 children develop TB each year, inexcusably, most anti-TB agents are not available in suitable pediatric formulations ^8^. While liquid formulations may be easy to administer to young children, they are bulky and more expensive, and some have unacceptable toxicity. Only recently have TB drug dosing recommendations been revised to reflect differences in the way children metabolize drugs and, until recently, most first-line drugs were under-dosed ^9^. Equally important, the last 50 years have only brought about the FDA licensing of two new drugs for TB treatment (rifapentine and bedaquiline) ( Figure 1). There is an urgent need for more effective and tolerable treatment of drug-susceptible and drug-resistant disease and latent TB infection as well as dosing strategies for children. Regimens that can be safely co-administered with antiretroviral therapy are also needed for the growing number of patients co-infected with HIV and TB.

There are some positive developments, though: after a long drought, new drugs are available, and new strategies for the treatment of latent disease and for regimen development in active disease are emerging. A three-month regimen of isoniazid and rifapentine has been approved for the treatment of latent infection in the United States ^10^, and even shorter courses of treatment are under study (AIDS Clinical Trials Group [ACTG] study 5279, NCT01404312).

New and old rifamycins are being repurposed at optimized doses. Clofazimine, an iminophenazine dye used to treat leprosy, has attracted renewed interest owing to its ability to target TB. Fluoroquinolones have been studied for shorter treatment durations of drug-susceptible disease, though phase III trial results have been disappointing thus far ^11^. Discussed in more detail below, there are novel drugs in new classes approved and/or in clinical trials, including bedaquiline, delamanid, pretomanid, AZD5847, and sutezolid. Used either separately or in novel combinations, these agents are anticipated to shorten and otherwise improve the treatment of drug-resistant and, possibly, drug-susceptible TB.

As important as the emerging new strategies for anti-TB regimen development are regulatory changes to expedite these efforts. Since the 1950s, TB clinical trials have consisted of addition to, or substitution of, an existing drug in a standard regimen. Traditional trial designs use TB cure without relapse as an endpoint, which requires observation for the duration of treatment and then 12–18 months of follow-up. Therefore, large phase III trials often take five or six years to complete and, with multiple compounds now in the pipeline, the development of a novel regimen using this model would require decades. The recent increase in global philanthropy directed at TB and the collaborations amongst major stakeholders have created opportunities to overcome bottlenecks and dramatically shorten the time to regimen development. For example, the Global Alliance for TB Drug Development, which is a not-for-profit product development partnership (PDP), is positioned to leverage a global network of public and private partners to advance TB drug development by developing drug combinations instead of individual drugs ^12^. Treatment shortening remains an important goal for the near future, as is the development of more tolerable regimens. Regimens consisting of novel drugs with activity against all TB, irrespective of resistance to drugs currently in use, are under discussion. Future goals in TB drug development also include the identification of relevant biomarkers to act as surrogate endpoints rather than waiting for relapse-free survival ^13^.

## New applications of existing drugs

### Rifamycins

Rifampicin is a cornerstone of TB treatment, largely because of its sterilizing ability (i.e. the ability to kill both rapidly dividing and dormant, less active mycobacteria). Matrix-assisted laser desorption/ionization (MALDI) mass spectrometry has shown that rifampicin efficiently penetrates sites of TB infection in lung lesions, and even accumulates in necrotic caseum ^14^. Surprisingly, the daily dose of 600 mg currently used is likely not on the optimal part of the dose-response curve, and higher doses may be needed to achieve treatment-shortening goals for drug-susceptible TB ^15^. High-dose rifampicin appears to be well tolerated, as shown in the RIFATOX study, for example, which evaluated 20 mg/kg versus 15 mg/kg versus 10 mg/kg of rifampicin ^16^; the HIGHRIF project, which is a series of four trials of high-dose rifampicin ( http://www.edctp.org); and an NIH-supported study in Brazil and Peru ( http://clinicaltrials.gov/ct2/show/NCT01408914). The PanACEA MAMS-TB-01 trial found that three months of dosing with 35 mg/kg of rifampin, in addition to standard isoniazid, ethambutol, and pyrazinamide, improved time to stable culture conversion over 12 weeks on liquid (though not on solid) media over the standard drug-susceptible TB treatment (median 48 versus 62 days; adjusted hazard ratio 1.75; 95% confidence interval: 1.21–2.55; P = 0.003) ^17^, without apparent toxicity from the higher rifampin dosage.

For HIV-infected patients requiring protease inhibitor-based antiretroviral therapy, rifabutin is generally recommended where available because of reduced potential for significant drug–drug interactions and is effective and safe in this setting ^18^. The ACTG study 5290 is currently comparing strategies of double-dose lopinavir boosted with ritonavir in combination with rifampicin-based TB treatment versus standard dosing with rifabutin (NCT01601626).

From the same class but with a longer half-life, rifapentine is being evaluated for both latent and active TB. In an 8,000-patient, international study from the TB Trials Consortium (TBTC) funded by the Centers for Disease Control and Prevention (CDC), weekly rifapentine at 900 mg with isoniazid 900 mg weekly for three months was non-inferior to nine months of isoniazid for the treatment of latent infection ^10^. The CDC guidelines were revised in 2011 to include this regimen ^19^. An even shorter regimen of rifapentine and isoniazid for one month in HIV co-infected patients is under study by the ACTG (A5279, NCT01404312).

The TBTC study 29 found similar rates of sputum culture conversion (i.e. from positive to negative for TB) at eight weeks in 381 smear-positive patients with TB using rifampin or rifapentine ^20^. Phase III trials with rifapentine for pulmonary TB are ongoing, with the launch of TBTC study 31 in late 2015. This phase III randomized clinical trial with 2,500 participants will investigate four-month rifapentine-containing (1200 mg daily) treatment regimens compared to the six-month standard-of-care rifampicin-containing regimen (NCT02410772).

Unfortunately, all rifamycins induce phase II and cytochrome P450 drug-metabolizing enzymes, resulting in significant drug–drug interactions when used with antiretroviral agents and other drugs. This presents a major obstacle to the treatment of HIV/TB co-infected patients with rifamycins, particularly those who require protease inhibitor therapy ^21^. Rifapentine was previously considered a less potent inducer of the metabolizing CYP3A isozyme than rifampin and therefore less likely to cause significant drug–drug interactions. However, data in healthy volunteers at clinically relevant doses using midazolam as a probe found rifapentine to be a more potent inducer of CYP3A than rifampin ^22^. Despite this, no clinically meaningful effect on efavirenz exposure was seen in 87 patients receiving a four-week course of daily rifapentine for TB prevention in ACTG 5279 ^23^. Rifapentine increased raltegravir exposure in a healthy volunteer study, but this was safe and tolerable; therefore, once-weekly rifapentine can be used in HIV-infected patients on raltegravir ^24^.

### Fluoroquinolones

Although not licensed for TB treatment, fluoroquinolones are often used in the treatment of drug-resistant TB and are also under study as first-line agents. Levofloxacin, moxifloxacin, and gatifloxacin are all active *in vitro* and in a mouse model ^25^. However, three recent phase III trials all failed to show non-inferiority to current standard-of-care regimens when investigating four-month treatment regimens containing moxifloxacin for drug-susceptible TB ^26– 28^. As the four-month regimens worked in a large majority of study participants, fluoroquinolone-containing regimens still warrant additional investigation and several studies are ongoing. TBTC study 31 will include moxifloxacin in one of its investigational treatment arms in combination with high-dose rifapentine, isoniazid, and pyrazinamide. The Global Alliance for TB Drug Development [ http://www.tballiance.org] is currently conducting a large, multi-country phase III trial investigating moxifloxacin in combination with PA-824 and pyrazinamide in the STAND Trial (NCT02342886).

Fluoroquinolones do not have significant drug–drug interactions with antiretroviral drugs, although adverse effects limit their utility in children and pregnant women. Moxifloxacin may also have overlapping toxicity with regard to prolongation of the QT interval.

### Clofazimine

Clofazimine is a drug currently used in combination to treat leprosy as well as in therapy for *Mycobacterium avium* infection. Clofazimine was first synthesized in the 1950s with the intent of treating TB, although its efficacy against TB was difficult to establish. With the discovery of rifampicin, clofazimine failed to find a place in TB treatment but is now being investigated as a possible treatment modality for treatment shortening and against drug-resistant TB. For example, TRUNCATE-TB will use an adaptive design to test several two-month drug-susceptible TB regimens including new and repurposed drugs (including high-dose rifampin, linezolid, clofazimine, delamanid, and bedaquiline). The ACTG is also developing a clinical trial for drug-susceptible TB based on preclinical work in the mouse model ^29^.

## New Drugs

### Bedaquiline (TMC207)

Bedaquiline is a diarylquinoline (a drug class not related to fluoroquinolones) with a novel mechanism of action which involves inhibition of the mycobacterial ATP synthase ^30^. It has potent activity against *Mycobacterium tuberculosis* isolates regardless of resistance but little activity against other common bacterial pathogens. Bedaquiline was approved by the FDA for the treatment of pulmonary multi-drug-resistant TB in December 2012, largely on the basis of phase IIb data ^31^. Multiple clinical trials are planned or underway to learn how best to use bedaquiline in drug-resistant TB treatment. From the TB Alliance, NC-005 is a two-month phase II study looking at pretomanid, bedaquiline, and pyrazinamide (NCT02193776). The STREAM trial is evaluating the effectiveness of two bedaquiline-containing regimens with the goal of developing an all-oral six-month regimen for multi-drug-resistant TB (NCT02409290). NIX-TB is an ambitious phase III trial utilizing bedaquiline, pretomanid, and linezolid for the treatment of extensively drug-resistant TB in six to nine months and was launched in early 2015 (NCT02333799).

Although bedaquiline is a substrate of the metabolizing enzyme CYP3A, problematic drug–drug interactions with antiretroviral agents are not anticipated because bedaquiline is metabolized only by CYP3A and does not induce or inhibit the enzyme. However, the drug has a very long half-life, and unanswered questions remain about long-term safety and tolerability. Concentrations of bedaquiline can be reduced by 50% or more with co-administration with rifamycins, but no significant effect was seen when given with efavirenz ^22^.

### Nitroimidazoles: pretomanid (PA-824) and delamanid (OPC-67683)

The next generation of nitroimidazoles shows promise for TB treatment. This includes pretomanid under development by the Global Alliance for TB Drug Development, and the Otsuka Pharmaceutical Company is developing a related compound, delamanid. Both have potent activity against drug-susceptible and drug-resistant TB *in vitro* ^32^. In mice, either drug in combination with rifampicin and pyrazinamide shortened TB treatment by at least 2 months ^33, 34^.

In patients with pulmonary TB, the addition of pyrazinamide to pretomanid or bedaquiline significantly increased the early bactericidal activity of both drugs ^35^. Early bactericidal activity studies measure decline in sputum colony counts per day among patients with sputum smear-positive pulmonary TB. A combination of pretomanid together with moxifloxacin and pyrazinamide in an early bactericidal activity study found that this three-drug combination was more effective than standard TB treatment (isoniazid, rifampin, pyrazinamide, and ethambutol), a first-ever finding in a two-week early bactericidal activity study ^36^. Though studies conducted in the first two weeks of treatment cannot predict a regimen’s ability to provide cure, these groundbreaking studies were greeted with great interest, and the next wave of trials is currently underway investigating the new paradigm of a universal regimen to treat patients with either drug-susceptible or -resistant TB.

Otsuka have completed a phase II, randomized, double-blind trial of delamanid in multi-drug-resistant TB patients receiving either 100 mg or 200 mg twice daily in addition to standardized second-line drugs. The addition of delamanid significantly increased the rates of sputum culture conversion at two months in patients with multi-drug-resistant TB ^37^. Results from this trial led to conditional approval from the European Medical Agency (EMA) in November 2013 for the use of delamanid in the treatment of multi-drug-resistant TB. The approved dose of delamanid is 100 mg twice daily in combination with an optimized background regimen. A larger phase III study of delamanid in adults with multi-drug-resistant TB including subjects on antiretroviral therapy is currently underway with results expected in 2017. Both delamanid and bedaquiline may cause prolongation of the cardiac QT interval, so there is concern about co-administration ^38^. The ACTG is conducting a study to address this issue ^39^. Delamanid appears to be void of clinically relevant interactions with antiretroviral therapy, which may make it an appealing agent to use in HIV/TB co-infected individuals ^40– 42^.

### Oxazolidinones

Oxazolidinones are protein synthesis inhibitors with a unique mechanism of action against TB ^43^. This class includes linezolid, sutezolid, and AZD5847. *In vitro*, linezolid is effective at doses of 1–2 µg/mL against 90% of clinical isolates ^44^ and works well in a mouse model ^43^.

Unfortunately, long-term administration of linezolid is associated with toxicities such as neuropathy and myelosuppression, but the drug has been evaluated retrospectively for the treatment of drug-resistant TB with encouraging results ^45, 46^. Ongoing clinical trials are planned to investigate linezolid in patients with multi-drug-resistant and extensively drug-resistant TB.

Sutezolid, a related oxazolidinone in development formerly from Pfizer but now licensed to Sequella, is superior in preclinical models and phase I bactericidal activity studies ^47^. Unlike linezolid, sutezolid appears to have sterilizing activity, a key factor in successful TB treatment. Addition of the drug to a standard regimen produced a 2-log drop in bacillary burden in a mouse model and appeared synergistic with rifampin ^48^; however, a two-week study in humans was unable to reproduce these findings ^49^. Unfortunately, the development of this compound has been complicated by licensing issues and remains extremely slow at present ^50^. Unlike linezolid, sutezolid does not appear to cause QT prolongation or bone marrow suppression; however, there are concerns of neurotoxicity and hepatotoxicity associated with its use.

AstraZeneca were developing an oxazolidinone, AZD5847, but the future of the drug is uncertain. A phase II early bactericidal activity study was completed in December 2013, but results have not been presented or published.

## Conclusions

After a long drought, the pipeline for new TB therapeutics is trickling. Caution still needs to be exercised with regard to identifying resistance to the novel drugs and ensuring that the optimized background regimen offers the maximum protection possible and there remains a bottleneck between preclinical and clinical development. However, there is a global commitment to major improvements and progress over the next decade towards the goal of eliminating TB as a global public health crisis.

## Abbreviations

ACTG, AIDS Clinical Trials Group; CDC, Centers for Disease Control and Prevention; HIV, human immunodeficiency virus; TB, tuberculosis; TBTC, Tuberculosis Trials Consortium.
